# Biological lipid nanotubes and their potential role in evolution

**DOI:** 10.1140/epjst/e2020-000130-7

**Published:** 2020-11-16

**Authors:** Irep Gözen, Paul Dommersnes

**Affiliations:** 1 Centre for Molecular Medicine Norway, Faculty of Medicine, University of Oslo, Oslo, 0318 Norway; 2 Department of Chemistry, Faculty of Mathematics and Natural Sciences, University of Oslo, Oslo, 0315 Norway; 3 Department of Chemistry and Chemical Engineering, Chalmers University of Technology, Göteborg, 412 96 Sweden; 4 Department of Physics, Norwegian University of Science and Technology, Hoegskoleringen 5, 7491 Trondheim, Norway

## Abstract

The membrane of cells and organelles are highly deformable fluid interfaces, and can take on a multitude of shapes. One distinctive and particularly interesting property of biological membranes is their ability to from long and uniform nanotubes. These nanoconduits are surprisingly omnipresent in all domains of life, from archaea, bacteria, to plants and mammals. Some of these tubes have been known for a century, while others were only recently discovered. Their designations are different in different branches of biology, e.g. they are called stromule in plants and tunneling nanotubes in mammals. The mechanical transformation of flat membranes to tubes involves typically a combination of membrane anchoring and external forces, leading to a pulling action that results in very rapid membrane nanotube formation – micrometer long tubes can form in a matter of seconds. Their radius is set by a mechanical balance of tension and bending forces. There also exists a large class of membrane nanotubes that form due to curvature inducing molecules. It seems plausible that nanotube formation and functionality in plants and animals may have been inherited from their bacterial ancestors during endosymbiotic evolution. Here we attempt to connect observations of nanotubes in different branches of biology, and outline their similarities and differences with the aim of providing a perspective on their joint functions and evolutionary origin.

The interior of a living mammalian cell contains membrane compartments with an incredible diversity of sizes and shapes. These membranes are not only structurally complex, they are also highly dynamic, and constantly flow and change in shape and topology. Here we focus on a particularly generic shape of biological membranes: membrane nanotubes, which are commonly observed structures in mammalians cells, and also occur in almost all other domains of life. The shape and dynamics of

[a] e-mail: irep.gozen@ncmm.uio.no
[b] e-mail: paul.dommersnes@ntnu.no nanotubes appear to provide advantages for biological necessities such as transport of materials, and signaling within or between cells. Some types of biological nanotubes were observed decades ago, others have been recently established. Nanotubes occur in a variety of organisms, in animals [1–3], plants [4–6], archaea [7–9], bacteria [10,11], and even viruses [12], where they have a variety of functions. This is astonishing; the membranes found in different cell types [13–16], even different organelles in a cell [17] possess distinct lipid compositions; yet one of the persistently reoccurring biological membrane forms is the nanotube. A large class of these tubes have diameters of around 100 nm, exactly the same as observed in model, i.e. in vitro, membrane systems. It is generally accepted that the morphogenesis of these biological nanotubes is governed by the mechanical properties of lipid membranes, which are universally liquid and flexible, i.e. they can be remodeled. In addition to mechanically extruded nanotubes, there is also a large class of tubes that form due to curvature inducing proteins. Since lipid membranes are ubiquitous in the realm of life, and the mechanisms governing their formation are straightforward, one can assume that nanotubes may have emerged independently in even remotely related organisms.

Nanotubular membranes have been studied in detail in many branches of life. In the following we attempt to connect the different types of previously identified forms of biological nanotubes, and discuss similarities between the emergence and functioning of the tubes in different organisms. We will describe the structural features of these biological nanotubes, their formation mechanisms, the associated functions, and possible involvement in evolution.

## 1 Formation of nanotubes in synthetic membrane systems

We first recall some of the basic properties of tubular membranes studied in synthetic model systems that are relevant for the discussion of biological nanotubes. Biological membranes consist largely of phospholipids and proteins, it is thus tempting to attribute the formation of membrane nanotubes to proteins. However, artificial phospholipid membranes free of proteins can be produced in the laboratory with mechanical properties very similar to their biological counterparts, which show the ability to create and maintain nanotubes with similar properties and dimensions. These model systems have been studied extensively (Fig. 1). The mechanical energy per unit area ($f$) of a membrane is a combination of curvature and surface tension energy [18]: $f = \frac{\kappa}{2}\left(c_1 + c_2\right)^2 + \sigma$ where $c_1$ and $c_2$ are the principal curvatures, $\kappa$ is bending modulus, and $\sigma$ the membrane tension. Since the membranes are fluid, there is no in plane elastic energy, only bending energy, and they can therefore take many shapes.

*Force induced tubes*: Applying a sufficiently strong point force (~10 pN) to a membrane results in the formation of a uniform tubular membrane (Fig. 1A). The transition is first-order, and the equilibrium tube radius is determined from the balance of bending and tension energies [19–21]. The equilibrium radius is:

$$r = \sqrt{\frac{\kappa}{2\sigma}}.$$

$\kappa$ and $\sigma$ have been estimated in different experimental systems. Typical values of $\kappa$ are 10–20 $k_\mathrm{b}T$ ($k_b T_\mathrm{room} = 4 \times 10^{-21}$ J) [22], and tension is typically in the range 0.01–0.05 mN/m [24], which results in nanotubes with diameters of the order 100 nm. Although the distribution of protein and phospholipid species varies among different organisms, most biological membranes have quite similar tension and bending rigidity as model systems, and therefore exhibit tubes of roughly the same diameters.

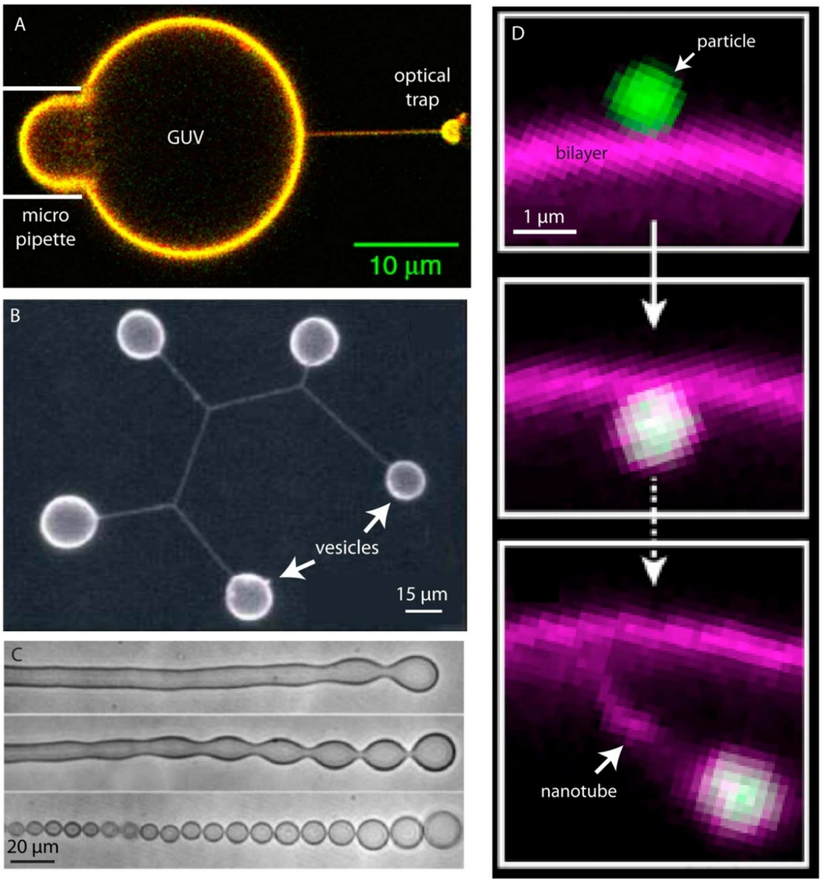

**Fig. 1.** Lipid nanotubes in model membrane systems. (A) Nanotube formation by applying a pulling point force to a giant unilamellar vesicle by means of an optical trap. Adapted with permission from reference [25]. Copyright 2019 Springer Nature. (B) Lipid nanotube vesicle networks formed manually using micro-needles. Adapted with permission from reference [28]. Copyright 2008 Springer Nature. (C) Pearling instabilities induced on membrane tubes by hydrophilic polymers with hydrophobic side groups along the backbone. Adapted with permission from reference [39]. Copyright 2001 American Physical Society. (D) Nanotube formation due to adhesion of microparticles to the membrane. Adapted with permission from reference [36]. Copyright 2017 Elsevier. (This figure is subject to copyright protection and is not covered by a Creative Commons license.)

Tip force-induced tubes will retract quite rapidly if the pulling force is removed. In model systems, the retraction speeds are typically in the range between 1 and 10 $\mu$m/s [21]. Force induced nanotube formation has been studied with optical tweezers [25], hydrodynamic flows [21], microfluidics [26], or micro-needles [27,28] (Fig. 1B).

It is possible for the two fluid membrane tubes to coalesce resulting in symmetric Y-junctions [29] with a 120 degree angle between tubes. The coalescence follows a zipper-like mechanism, in which two tubes fuse to form a single, unified tube [30]. Since the phospholipid membranes are fluid, any gradient in membrane tension will create a flow of membrane from lower to higher tension regions, i.e. Marangoni flow [31]. This type of flow has been demonstrated and used to transport molecules between vesicles connected with membrane nanotubes [32]. This dynamical property of lipid membranes is fundamental for biological systems. For example, Golgi networks inside cells, which package proteins inside membranous compartments before they are sent to their destination, have lower tension than the Endoplasmic Reticulum (ER), to which it has contact sites. Golgi tubes that fuse with the ER nanotubular network are drawn into the ER by tension driven membrane flow [33]. The nanotubular network of ER will be discussed in more detail in the following sections.

*Tubulation by curvature-inducing molecules or particles*: Long uniform tubes can also be created by the adsorption of curvature inducing amphiphilic molecules to the model membranes [34] (Fig. 1C), adsorption of nano- [35] and micro-particles [36] (Fig. 1D), or curvature inducing proteins [37]. These tubes tend to have a broader distribution of diameters; in particular, the protein-induced tubes have diameters down to 20 nm. The tube formation can be simply induced by the molecules imposing a specific curvature, which requires a rather high concentration of molecules/proteins. The tubulation can also be caused by an asymmetry in the effective area of the two leaflets of a bilayer membrane, which induces a global spontaneous curvature constraint [38]. Compression of membranes can also induce a similar effect, causing inward tubulation in vesicles [39].

## 2 Formation of nanotubes in biological cells

Biological nanotubes are often highly dynamic, i.e. they can form and disappear rapidly, such as mitochondrial networks or stromule tubes, with dynamics quite similar to what is seen in model membrane systems. There exist, however, nanotube structures that are much more static, one example is the T-tubules in heart cells [40,41].

Any force pulling on the membrane in the order of ∼10 pN, will result in tube formation [42–44]. For example in Golgi complex, the tube formation is driven by motor proteins [42,45]. The biogenesis of the ER is enabled by proteins, e.g. atlastin, which tether the membrane to the microtubules and allow the formation of ER membrane extensions using microtubule motor activity [46,47]. Mitochondrial networks form in a similar manner, mediated by membrane contact points to the ER which promote the coalescence and division of mitochondrial membranes [47,48].

The nanotube formation can occur due to purely mechanical instabilities and do not necessarily require the activity of molecular machinery. This ability provides enormous benefits for dynamic biological processes featuring lipid nanotubes as key structures. For example, the cellular organelles which have a sufficient source of lipids, but cannot rapidly synthesize proteins, can exhibit tube formation. The capability of efficiently forming nanotubes may be important during cellular migration (migrasome [49]), or as a part of immune response (stromule [50]).

The regulation of nanotube morphology and stability may still require proteins. A prominent example are BAR proteins, which can both sense and induce curvature on membranes [37,51]. Dynamin is another example: it can transform membranes into nanotubes by polymerizing as a helical structure around the membrane, and also has the ability to induce membrane fission [44]. While dynamin induces curvature on the membrane, it has also been shown that a curved membrane induces polymerization of dynamin [44]. This finding may be interesting from an evolution point of view, as it indicates that the polymerization of dynamin could have occurred for the first time using lipid nanotubes as templates. Amphiphiles, e.g. fatty acids or phospholipids, are thought to have existed on the early Earth much earlier than proteins [52].

## 3 Nanotubes in bacteria, archea and viruses

Biological nanotubes were first discovered in Eukaryota, and their study in this context is a well-developed field by now. In the past decade it has become clear that biological nanotubes also exist among bacteria and archaea [7,8], and even in viruses. In bacteria, which primarily communicate through secreted extracellular compounds, nanotubes enable direct, contact-dependent exchange of molecules [10,11]. Since bacteria have cell walls, the donor bacteria utilize enzymes which can hydrolyze the cell wall of the receiving bacteria before soft matter lipid nanotubes can reach to the plasma membrane of the recipient bacteria [11]. The donor delivers the enzymes to the recipient through the nanotube which eventually localize at the nanotube contact sites. Through the pierced cell wall, the nanotubes are efficiently extruded and prolonged [11]. It has been shown that some bacteria extend nanotubes like "tentacles" to prey on other bacteria, simultaneously delivering toxin and acquiring beneficial cargo [53]. This suggests that bacterial nanotubes involve quite sophisticated mechanisms, which opens up the possibility that they have been passed on to eukaryotic cells by symbiogenesis.

It appears that the bacteria have two types of nanotubes. One type is the short and tunneling (open-ended) tubes that connect them to nearby bacteria (Fig. 2A). The other type occurs at low bacterial density, where the nanotubes seem to grow from the surface and display more complex branched morphologies [54] (Fig. 2B). These tubes that are reminiscent of roots can form relatively rapidly (minutes) and can extend several tens of micrometers away from the bacteria. The extended tubes eventually connect to other bacteria, leading to intercellular networks. The extending nanotubes exhibit consecutive constricted segments with continuous lumen, quite similar to the pearling instabilities observed in tubular membranes [39] (Figs. 2C and 1C). The tip segments can bud off at the tube ends, and become independent nano-sized membrane vesicles, a process commonly referred to as "tip shedding" (Figs. 2D–2E). Extracellular membrane vesicles which form by budding from bacteria have previously been shown to transfer genetic information over long-distance via shuttling RNA between cells [55]. Shedding of nanotubes leading to the formation of extracellular vesicles has also been observed in archaea [7] (indicated by the white arrow in Fig. 2F).

In 2013, membranous tunneling nanotubes were observed in PRD1 viruses with internal membrane compartments [12] (Figs. 2G–2J). The vesicle inside the viral capsid protects the double stranded DNA of the virus, and it has been hypothesized to be the key player in genome delivery. Before the genome translocation occurs in a few minutes, the membrane vesicle bends with the help of specific membrane proteins, which comprises 50% of the membrane volume. A large fraction of the vesicle transforms into a nanotube which extends out through a unique aperture in the virus capsid, and penetrates the host bacterial cell envelope. This process bears some resemblances to the tubes extending from bacteria (Figs. 2B–2E).

## 4 Nanotubes in eukaryotes

Cellular structures associated with nanotubes and nanotubular networks in eukaryotes are abundant. Prominent examples are mitochondrial networks, the ensoplasmic reticulum (ER), T-tubules, tunneling nanotubes (TNTs) – specifically cytonemes and migrasomes–, and stromules. T-tubules, cytonemes and migrasomes are physically connected to the plasma membrane, while ER, mitochondria and stromules are independent intra-cellular organelles.

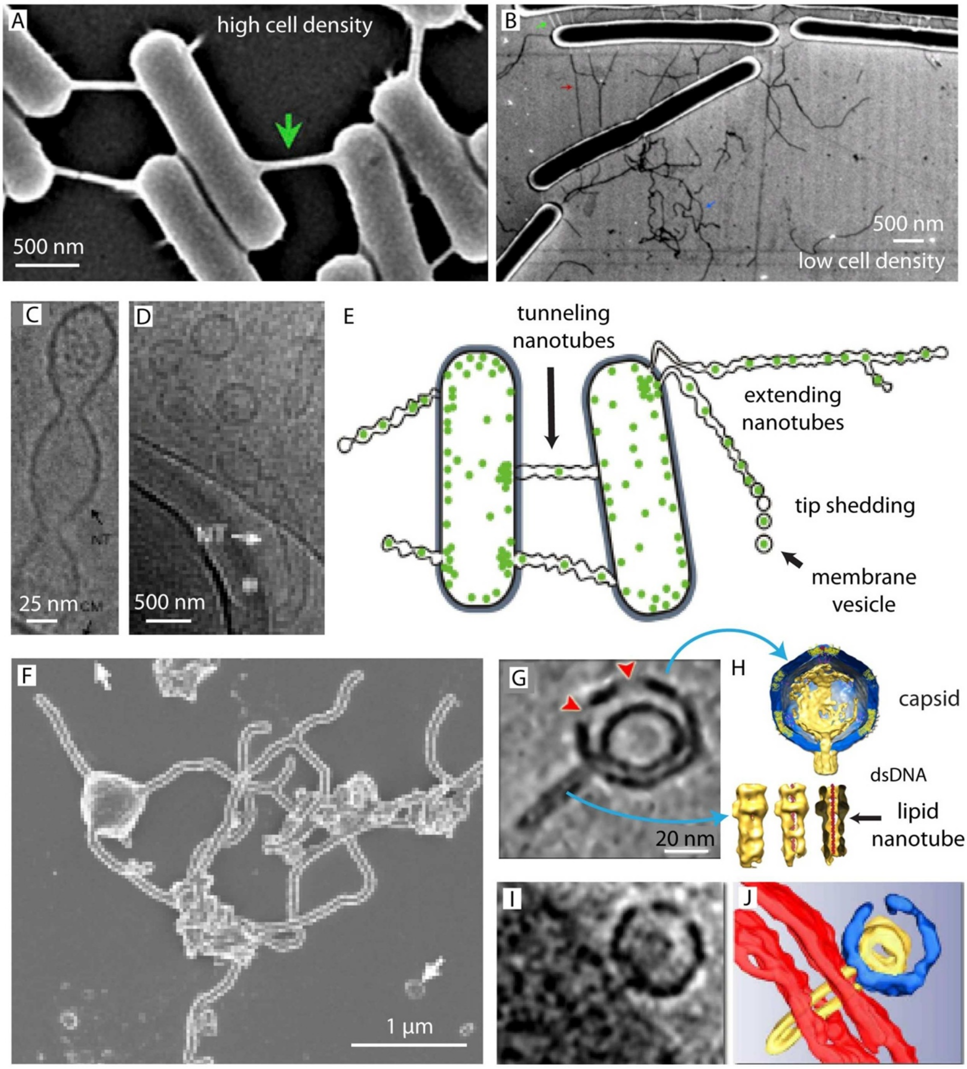

**Fig. 2.** Examples of biological nanotubes in bacteria, archaea and viruses. (A) TNTs among bacteria at high cell density. (B) Root-like lipid nanotubes emerging from bacteria at low cell density. (C–D) Pearling-like instabilities in bacterial nanotubes leading to tip shedding. (E) Drawing depicting the extending nanotubes and tip shedding. (A–E) Adapted with permission from reference [54]. Copyright 2016 Elsevier. (F) Nanotubular networks in archaea. Adapted with permission from reference [9]. Copyright 2020 Nature. (G–J) Lipid nanotubes (yellow) observed in viruses. In (I–J) the penetration of the viral nanotube into the host bacterium has been shown. Viral capsid is shown in blue color. Adapted with permission from reference [12]. Copyright 2013 PLoS.(This figure is subject to copyright protection and is not covered by a Creative Commons license.)

**Mitochondria** (Fig. 3A), in which the biochemical processes of respiration and energy production take place, exhibit a diverse range of morphologies from fragmented structures to intact dynamic networks continuously undergoing fusion and fission events (meso- and hyper-fused states) [56]. A membrane potential in the order of 20–180 mV has been measured throughout the mitochondrial networks [57]. This potential, which is generated as a part of ATP synthesis, is critical for fusion. If the cell is in a stressed state, the fusion of the mitochondrial tubules is induced by means of this membrane potential [57]. The possible evolutionary benefits of mitochondrial networks will be discussed in the last section.

**The ER** (Fig. 3B) has crucial functions in almost all eukaryotic cells, including protein and lipid synthesis. It was recently discovered, owing to super-resolution microscopy, that the structures previously thought to be flattened membrane sheets at the peripheral ER, are in fact densely packed nanotubes [59]. The Golgi complex, which is bridged to the nucleus by the ER, is not considered to be a tubular organelle. However, research shows the existence of short nanotubular structures between the Golgi stacks [60] as well as intermediate nanotubes between the ER and Golgi that comprise 20% of the transport carriers between the two organelles [61].

The existing theories on the origin of the ER assumes that its initial formation starts with invaginations of the plasma membrane either from the outside inwards during the internalization of a prokaryotic organism [62], or from the inside outwards by forming protrusions turning into blebs around the organism [63]. The multiple invaginations later contact at multiple locations inside the cells and transform into the complex ER morphology in 3D. The tubular ER networks play a role in the formation of the lipid droplet organelles. Lipids, condensed in the tubes, grow into phospholipid covered droplets. It seems that the condensation of lipids may be induced simply by the curvature of the membrane [64]. In the last section, we will talk about the possible origins of ER tubular networks considering the organelle's exclusive features.

**T-tubules** (Fig. 3C), i.e. transverse tubules, extend from the plasma membrane inwards, towards the interior of skeletal and cardiac muscle cells, close to the neuromusular junctions [40,41]. They enable the transmission of action potential and forceful contraction of muscle cells [68]. The initial observation of T-tubules was made 1800s by Retriuz and was later closely investigated by Tiegs and others in the 1920s [69]. The nature of the T-tubules was redefined with the help of improved imaging techniques, especially through 3D visualization enabled by the advent of laser scanning confocal microscopy in the early 2000s [70]. T-tubules accommodate electrical signals, where the nanotube can act as a nano-neuron. A well-known example is the transmission of action potential, which is necessary for the function of the skeletal and cardiac muscles. For example, for mammalian cardiac cell function, many of the proteins involved in cellular $Ca^{2+}$ cycling appear to be concentrated at the T-tubules [68]. Propagation of action potential requires rapid changes in membrane potential. Direct measurements of sarcoplasmic reticulum (SR), which is the adjacent structure to T-tubules that store the $Ca^{2+}$ in muscle cells, by tracking $Ca^{2+}$ spikes in rat cardiac myocytes indicate the transmitted action potential to be around $-60\,\text{mV}$ [71]. Considering that the T-tubules are adjacent to SR and transmit the signal to them through high voltage-gated membrane channels (L-type) embedded the T-tubule membrane [41], it is conceivable that the T-tubules are capable of transmitting an action potential of $-60\,\text{mV}$.

**Tunneling nanotubes (TNTs)**, discovered in 2004 by Gerdes et al. [1], are a general term for cell membrane protrusions interconnecting animal or other cells. In 1987, Locke reported on nanofilopodia (ø $\sim$100 nm) which arose at the edges of the insect cells, extended and eventually adhered to distant neighbouring cells [72]. Although nanofilopodia are structurally TNTs, a specific function was not described in the study by Locke.

TNTs are used for inter-cellular communication under stress conditions, and the transport of small molecules, vesicles and even organelles, e.g. mitochondria [73] is possible from one cell to another through the TNTs. Tunneling nanotubes are also connected to disease. It has been proposed in one example study that by bridging

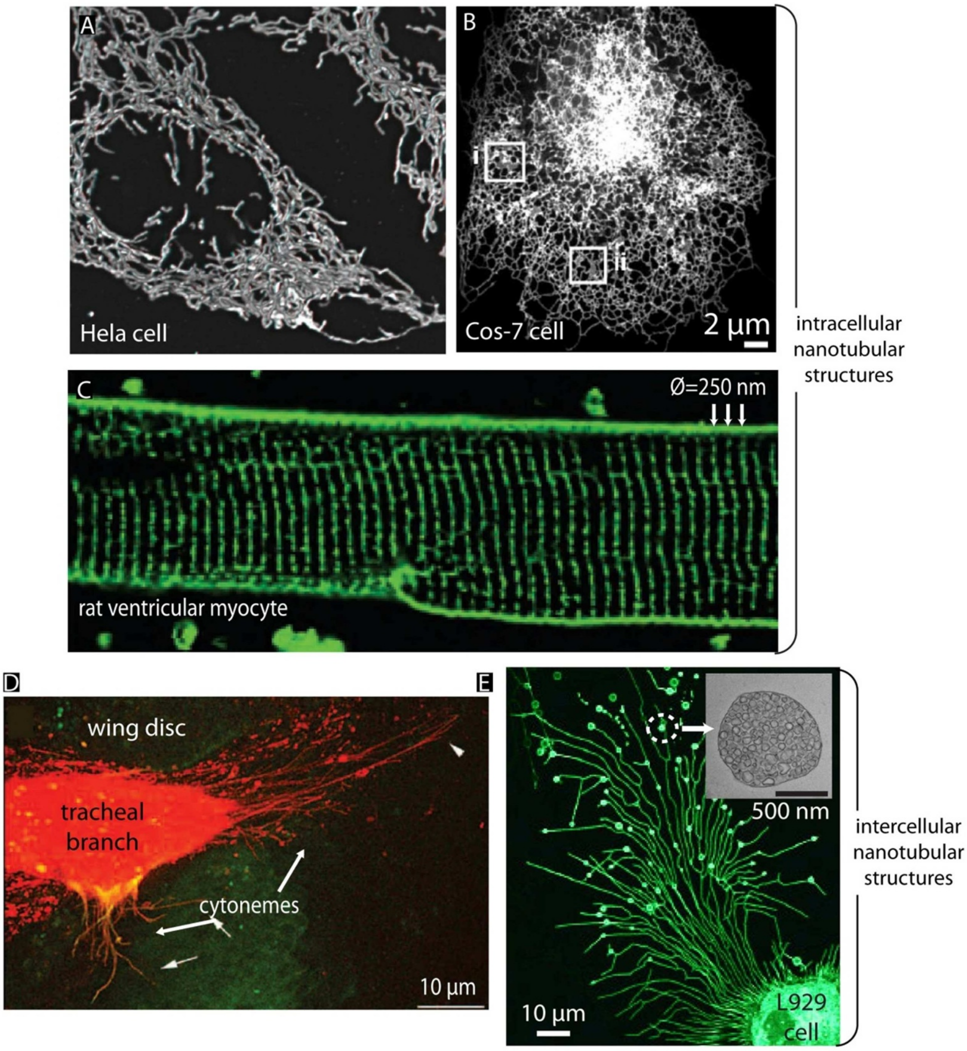

**Fig. 3.** Biological nanotubes in eukaryotes. (A) Mitochondrial networks inside a HeLa cell. Adapted with permission from reference [65]. Copyright 2009 Elsevier. (B) ER networks inside Cos-7 cells. Adapted with permission from reference [59]. Copyright 2016 The American Association for the Advancement of Science. (C) T-tubules in a rat ventricular myocyte. Adapted with permission from reference [40]. Copyright 2013 Oxford University Press. (D) Cytonemes. Adapted with permission from reference [66] Copyright 2014 The American Association for the Advancement of Science. (E) Migrasome generated by a L929 cell. Inset shows a transmission electron microscope image of the lipid pockets. Adapted with permission from reference [67]. Copyright 2018 Springer Nature. (This figure is subject to copyright protection and is not covered by a Creative Commons license.)

primary neurons and carrying Tau fibrils, a key protein in Alzheimer's disease, TNTs contribute to the prion-like propagation of Tau assemblies [74].

**Cytonemes (insects)**: A special type of TNTs are cytonemes [66] (Fig. 3D). These cellular projections specifically transport signaling proteins in imaginal disc cells found inside larva of insects that undergo metamorphosis [2,3]. Their radius is in the range between 0.1 and 0.5 $\mu$m. Cytonemes were observed for the first time in 1995 by Miller et al. in sea urchin cells and referred to as "thin filopodia" [75]. The name *cytoneme* was coined a few years later in 1999 by Kornberg et al. [3]. Cytonemes transport morphogens [2], the signaling proteins that regulate the patterned development of insect embryos and tissues.

**Migrasomes** (Fig. 3E), discovered in 2015, are exosome-like pockets emerging from nanotubular networks formed during cell migration in vitro. It was observed that lengthy, branched, plasma membrane-connected lipid nanotubes attached to exosome-like vesicles, altogether termed the migrasome, appear at the rear end of migrating cells [49,67,76,77]. So far, the function of migrasomes has been proposed to be facilitating cell-to-cell communication. They have also been recently detected in human serum [78]. Ongoing research efforts are focusing on identifying migrasome-specific markers and potential associated functions. Migrasome formation is migration dependent, although the function of migrasome is not directly associated with enabling individual cells to migrate. While cells translocate, they leave behind actin-rich lipid nanotubes, from which pockets containing small vesicles grow. The nanotubes disconnect from the migrating cell over time, leading to release of vesicular contents into the extracellular space which are directly taken up by the cells in the vicinity. This communication mechanism appears to be exclusive to migrating cells. Migrasomes have distinct compositions compared to extracellular vesicles, exosomes, and intracellular organelles. They have been proposed as providers, enabling spatiotemporal chemical information for cell–cell communication.

Migrasomes have been also shown to be essential for organ morphogenesis during the embryonic development of zebra fish [77]. During this process, migrasomes release ligands and proteins at defined locations of embryos to induce formation of organs. One of these proteins, CXCL12, has the ability to induce directed chemotaxis in nearby responsive cells. This suggests that migrasomes serve here as local chemoattractants.

**Stromules (plants)**: Plants have many organelles, such as mitochondria and the ER that are similar to animal cells, but also feature organelles that are unique to plants (and algae) for example plastids. Plastids are hypothesized to be evolved from cyanobacteria through endosymbiosis, have their own DNA, and perform essential tasks related to storage and photosynthesis. Long nanotubular extensions called stromules (Fig. 4) were observed more than a hundred years ago (Gottlieb Haberlandt, 1888 and Gustav Senn, 1908) in plastids. These tubes are somewhat larger; their diameter is in the range between 350 and 800 nm and can extend up to 200 $\mu$m. They exhibit strong similarities with the comparatively thin, mechanically extruded membrane nanotubes. It is known that the stromules can be formed by anchoring the plastids to other plastids or organelles like the nucleus or the ER network. During cytoplasmic streaming, the forces that act, among other organelles, on the plastids, cause the extrusion of the stromules at anchoring points, in a fashion very similar to anchored model membrane vesicles observed in hydrodynamic flows. When stromules detach from an anchoring point, they retract spontaneously and quite rapidly at a velocity of about 0.5 $\mu$m/s [50], which is a clear indication that they are formed mechanically, and not by the (much slower) action of proteins. Stromules have indeed been reported to form in association with ER nanotubular network in plant cells [79], and display quite symmetric Y-junctions similar to the ones in ER, or in artificial nanotube networks. If the stromules are formed by adhering to the ER network, this could be equivalent to the one-dimensional wetting phenomenon as recently proposed for membrane nanotubes adhering to fibers [80]. The speed of the retraction of stromules is an order of magnitude slower than in model membranes [21], this is consistent with the stromules having relatively low tension, but it could also be due to the higher viscosity of the cytosol. The larger radius of stromules may also be due to the double membrane structure of plastids, which could results in a higher bending modulus. Another reason may be the low membrane tension of plastids. It is known that plastids have mechanosensitive channels similar to bacteria, which protects them against osmotic shocks [81], it is possible that these channels have a role in maintaining low tension while stromules are extruded.

The biological function of stromules seems to have been overlooked for a century. They were in a sense rediscovered in 1997, when the stromules were shown to act as protein transporters between two stromule-connected plastids [82]. It is established that plastids like the chloroplasts, have originated from bacteria, are central to the immune system of plants, where they work in conjunction with the nucleus and other organelles to combat invading pathogens. In the case of a microbial infection, chloroplasts-to-nucleus retrograde signaling is initiated [83]. In order to achieve this, the plastids sense the invading pathogens and send signaling molecules to the nucleus, which triggers the immune response. An increasing amount of evidence indicates that specifically the stromules are an integral part of the plant's immune system. In 2015 it was discovered that the stromule has a central part in the stress-response mechanism [84], and that physical co-localization of chloroplasts (and nucleus) is not necessary. Stromules enhance programmed cell death and may facilitate its progression during an immune response [84]. Transport of signaling proteins and reactive oxygen species through stromules has been shown [4]. Specifically, hydrogen peroxide ($H_2O_2$), utilized by bacterial colonies for signaling/quorum sensing [85], acts as a signaling molecule and is transported through the stromules to the nucleus [4].

Stromules also produce small vesicles through tip-shedding [50]. While it is presently not exactly known which role these vesicles play, the strong similarity with tip-shedding in bacteria, where extracellular vesicles are used for signaling transport (Figs. 2D–2E), is worth noting. To this date the full extent of signaling through stromules, i.e. chemical or electrical, remains unclear, but the evidence suggests that the transmission of action and variation potential through stromules is a possibility [86]. Plant cells also contain ER network nanotubes, and stromules have been observed to interact with these networks [79]. The ER network of plant cells has a similar structure to the ER networks present in animals, and will be discussed below.

## 5 Evolutionary aspects

Membrane nanotubes may have played important roles in evolution, both at an early stage in the origin of life, and as inherited structures in symbiotic evolution. Endosymbiotic theory hypothesizes that the organelles of eukaryotic contemporary cells have descended from prokaryotes. Briefly, an initially independent, living single-celled organism such as bacteria or achaea, was taken up by another for a mutual benefit (endosymbiosis) and over time evolved to an organelle with a specific function. Prominent examples are mitochondria [104] and chloroplast [105]. Recent evidence has revealed that prokaryotes, bacteria for example, utilize lipid nanotubes for a variety of essential tasks such as plasmid transfer and quorum sensing. It is conceivable that the formerly free-roaming bacteria established and utilized the same membrane structures also inside their host cells. Figure 4A shows the tree of domains in which the observed membraneous nanotubes (colored boxes) and membraneous organelles (asterisks), have been indicated.

Organelles which are hypothesized to be resulting from the endosymbiosis, depict tubular nature. One example is mitochondrial networks. Although the particular role of mitochondrial networks is not yet fully understood, a few potential advantages have been proposed by Hoitzing et al. [56]: (1) when the tubular network form of the

**Table 1.** Dimensions and structural properties of biological lipid nanotubes.

| | Inter-cellular | | | Intra-cellular | | | |
|---|---|---|---|---|---|---|---|
| | Tunneling nanotube (TNT) | Migrasome | Cytoneme | T-tubule* | Endoplasmic reticulum (ER) | Mitochondria | Stromule |
| Diameter (nm) | 50–200 [1] [87] | 125 [78] | 100–200 nm [3,88] | 20–35 nm (Skeletal T-tubule) [89] 20–450 (Cardiac T-tubule) [70] | 20–50 [90–92] | 100–530 nm [93,94] | 100–850 [95] [82,97] |
| Membrane lamellarity | Single bilayer | Single bilayer | Single bilayer | Single bilayer | Single bilayer | Double bilayer?⋆ | Double bilayer?⋆ |
| Internal cortex | + [98] | + [49] | + [99] | ?† [100] | – [101] | ?‡ Mitochondrial micro-networks [102] | – [103] |

*Connected to the plasma membrane.
†The actin cytoskeleton is important for maintaining the structural integrity of the T-tubule however it is not well known how exactly T-tubules interact with actin filaments.
‡It is well-established that the micron-size mitochondrial networks attach to actin and microtubules, but currently there is not much information on how exactly mitochondrial nanotube networks interact with the actin cortex.
⋆The stromules and mitochondria are enveloped in double lipid bilayer organelles so the nanotubes emerging from them are expected to contain double membranes. However if the adhesion between the two bilayers is weakened, it is possible that single bilayer stromule or mitochondrion tubes are formed.

organelle is active, there is no need for a regulated selective process to remove dysfunctional components of the organelle for quality control. The dysfunctional parts can efficiently be separated from the network by fission as part of an initial screening; before they are eliminated by the cells in a programmed manner. (2) As the networks become gradually more interconnected, the fast-diffusing components on the network, e.g. proteins, will rapidly explore the new available spaces. The networks influence the diffusion rate of proteins non-linearly. (3) The fragmented mitochondria are more vulnerable to fluctuations in membrane potential, they can depolarize. The elongated tubes would increase the robustness of the organelle by buffering the sudden changes in membrane potential, eliminating biochemical and physical perturbations. The proposed advantages discussed here are tightly connected to the materials properties of membranous networks and may therefore not be limited to mitochondria.

The ER could also be originating from a prokaryote. One of the exclusive features of the ER is the continuous flow of the membrane to ER's peripheries indicating a tension gradient. In fact, the tension gradient throughout ER networks has been experimentally measured [33,106]. The ER could be derived from a prokaryote, which would be possible if two different species of prokaryotes with distinct membrane tensions become in contact and later transform into intracellular organelles, e.g. Golgi networks and the ER. A similar idea has been formulated by Gould et al. for the origin of endo-membranes between archaea and bacteria [107], which are intracellular vesicles highly associated with the ER. Mixing of two different species of bacteria (one gram positive, other gram negative) [108] results in formation of tunneling nanotubes and transport of molecules between the bacteria. This shows how robust nanotube communication is in the bacterial world, and shows that the nanotube connections between different organelles (mitochondria, plastids, ER, golgi) could have been inherited from bacterial ancestors.

One other nanotubular structure which could have potentially evolved from prokaryotic organism is the stromule. The closest living relative to plastids is a cyanobacteria found in the alkaline Lake Alchichica in Mexico. Nanotubular extensions and connections are seen in cyoanobacteria from this lake (Fig. 4B) [109], which suggests that plastid stromules/nanotubes could potentially have evolved from these bacterial nanotubes. Vincent et al. recently discussed the similarity between nanotubes in mitochondria and bacteria [110] They suggest that nanotubes are evolutionarily conserved, with one important role being horizontal gene transfer, e.g. transfer of genes between organisms, which is an important process in bacterial colonies. It is quite conceivable that also other tubular organelles in eukaryotic organisms, such as the ER network, could have inherited nanotubular transport and communication mechanisms originally developed by prokaryotic organisms.

Bacterial membrane nanotube most likely play an important role in gene transfer between bacteria, however there might be many other functionalities as the tubes work as communication and sensing organs of the bacteria. Perhaps the simplest functionality one could think of is to sense the presence of other bacteria. It was observed that long branching nanotubes grow from bacteria when the bacterial density is low [54], this could be a way for bacteria to make decisions such as whether to divide or not. Plastids like chloroplast sometimes form dense assemblies inside cells, which suggest that division and growth must be finely regulated to fit the space of the host cell. Moreover, during growth of the cell the chloropast density might suddenly drop, thus it has been suggested that stromules (Fig. 4C) are used by plastids to sense tension and overall concentration of other plastids [111], using mechanosenstive channels that are in fact inherited from prokaryotes. It is therefore plausible that plastids' density detection by stromule nanotubes could have been inherited from similar mechanism in cyanobacteria.

Plastids in plant cells communicate with the nucleus via reactive oxygen species (ROS) signaling, in particular by means of hydrogen peroxide. During pathogen infection, numerous stromules connect plastids and nucleus, and the concentration of $H_2O_2$ is observed to increase in the nucleus [5,84]. The plastids detect the intruder and send signals to the nucleus, a very important mechanisms of the innate immune system of plant cells. Although $H_2O_2$ is well known for its anti-microbal oxidizing action, microbes do produce $H_2O_2$ by themselves and multicellular microbal communities [114]. Since ROS are natural byproducts of both plastids and mitochondria, it is not impossible that the stromule-mediated ROS signaling in eukaryotic cells is inherited from the cyanobacteria, essentially mimicking stromules attached to the nucleus instead of another bacterium. One can then also speculate that this was one of the original evolutionary advantages of co-existence, i.e., the stromules were used as a signaling network to fight viruses or other bacteria. Plastids such as chloroplast are in fact key defence organelles in plants, and work as sites for production of pro-defence signals to the nucleus [115].

A recent study reports that the SARS-CoV-2 (COVID-19) virus causes the rapid growth of numerous nano-filopodia (ø:100–200 nm) after infecting animal cells. This happens when the virus hijacks a particular intracellular protein, CK2, and forces the cell to transform its shape. As a result, the cell grows TNTs, at the tip of each of which viruses emerge, ready to infect adjacent cells [116]. Similar effects have earlier been reported for other viral diseases [117].

The interaction between plants cells, plastids and viruses is particularly intriguing, and must have played a role in the evolution of plants. The role of stromule nanotubes in this interaction is, however, not straightforward: while stromules are used as communication lines between plastids and nucleus using signaling molecules like ROS (in particular $H_2O_2$) or NRIP1 [115], it is also known that viruses can trigger the formation of stromules and use the stromules to infect plant cells. Stromules can connect to both the plasma membrane and the nucleus, and it has been suggested that DNA viruses can use these stromule pathway to go directly to the

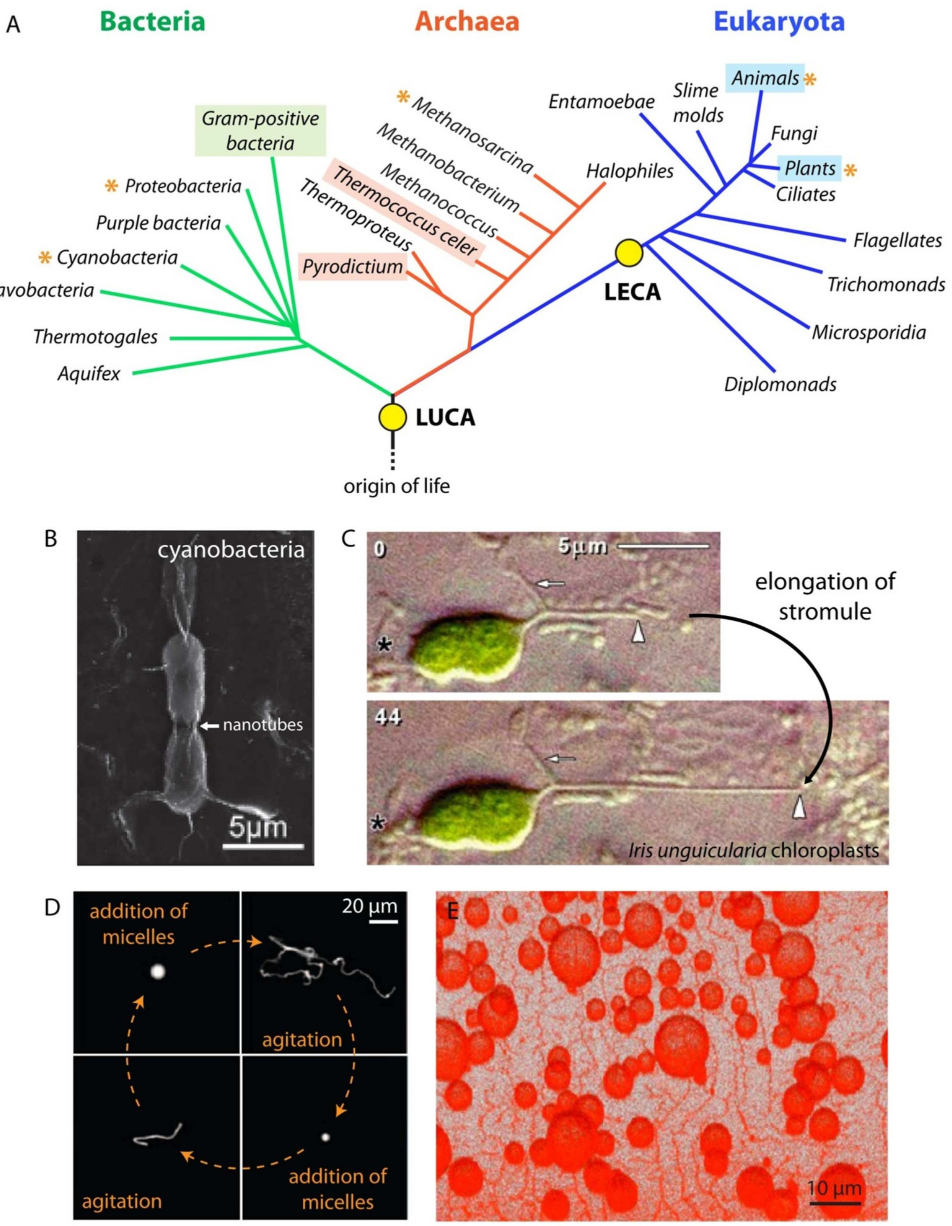

**Fig. 4.** Nanotubes in the context of evolution. (A) Tree of domains showing major branches: Bacteria, Archaea, and Eukaryota. The occurrence of membraneous nanotubes in each domain has been indicated with colored boxes, and membraneous organelles with asterisks. LECA: last eukaryotic common ancestor, LUCA: last universal common ancestor. (B) Cyanobacteria with tunneling nanotubes. Adapted with permission from reference [109]. Copyright 2012 The American Association for the Advancement of Science. (C) Stromules. Adapted with permission from reference [50]. Copyright 2005 Springer Nature. (D) Microtubular vesicles going through two cycles of growth and division. Adapted with permission from reference [112]. Copyright 2009 American Chemical Society. (E) A lipid nanotube network formed with autonomous transformations of a lipid reservoir on a mineral-like surface. Adapted with permission from reference [113]. Copyright 2019 American Chemical Society. (This figure is subject to copyright protection and is not covered by a Creative Commons license.)

nucleus without ever entering the cytosol [118]. Therefore the stromule nanotubes can be a double-edged sword, they play a role in defending against invaders, but can also be used by the invader. Virus infection through tunneling nanotubes (TNT) in mammalian cells has been studied quite extensively since the discovery of TNT. Rustom et al. have noted that stress ROS trigger formation of tunneling nanotubes in mammalian cells [119] and $H_2O_2$ in cell cultures induces the formation of TNT between different cells [120]. They made a connection with ROS-regulated transport through desmotubules (membrane nanotubes with diameters of 15–20 nm) connecting the ER of two neighbouring plant cells [121].

There is ample evidence suggesting that lipid bilayer membranes and membrane proteins co-evolved, i.e. the formation of membrane proteins required the presence of lipid membranes [122]. It has also been suggested that water soluble proteins evolved from membrane proteins [123]. It is therefore interesting to consider the possible role of membrane nanotubes in the evolution of proteins and protein assemblies.

*Origin of viruses*: Bacteria-derived membrane nanotubes surround protein pockets containing RNA or DNA, which later shed and form individual extracellular membrane vesicles to be transferred to other bacteria (Figs. 2D–2E) [54]. Nucleic acid transporting membrane vesicles exist in all domains of life and probably predated the last universal common ancestor (LUCA). This has led to the *escape hypothesis* for the origin of viruses [124] the virus capsid is proposed to have evolved from curvature forming proteins at the membrane of some ancestral RNA cells. Eventually, the replicating RNA producing the protein capsid was trapped in the capsid it produced, and the virus was formed. Present-day retroviruses operate in a similar manner, the virus capsid assembly occurs directly on the cell plasma membrane before escaping the cell [125]. This also suggests that the evolution of viruses may be linked to membrane nanotubes, i.e. proteins may have evolved to adapt to the tubular membrane shape, which subsequently formed protein-membrane capsules for transport of the genetic material. The recently discovered virus membrane nanotubes illustrate the close interaction between the capsid and the membrane nanotube (Fig. 2G). The capsid of filamentous viruses found in plants, e.g. tobacco mosaic virus, also bears some resemblance to protein self-assemblies that drive nanotubulation in membranes. It is noteworthy that the diameter of viruses and membrane nanotubes are very similar. The viruses of today are known to induce tube formation both in biological cells and model membranes [126].

*Evolution of tubular protein filaments*: FtsZ is a ring-forming protein found in archaea, bacteria, mitochondria and plastids. It plays an essential role in the cell division by forming rings that shapes the membrane and performs fission, in a similar fashion to dynamin. FtsZ rings in tubular model membranes gather and self-assemble [127], most likely due to the membrane mediated interactions between the individual protein rings [128]. In fact, FtsZ is a bacterial homologue of tubulin [129] which polymerize into microtubules. This puts forward the possibility that the tubular protein structures like microtubules could have evolved from the membrane nanotubes, i.e. the membrane nanotubes served as templates for protein self-assembly. It was previously thought that bacteria acquired tubulin from eukaryotes, however it is now known that there exist tubulin homologues in bacteria, that do form tubular filaments [130]. This suggests that tubular protein filaments evolved in archaea and bacteria long before the existence of eukaryotes.

The recent findings revealed the presence of membranous compartments with specific functions in archaea and bacteria [131]. This means that the membrane-enveloped intracellular compartments, the functions of which have previously been associated exclusively with eukaryotes, have ostensibly existed in prokaryota. This establishes the possibility that membranous organelle-like units and nanotubes could have existed in the last universal common ancestor (LUCA), and might have originated earlier than endosymbiosis (Fig. 4A).

The formation of tubular vesicular structures without membrane bending components has been previously studied in the context of the origin of life. Szostak and coworkers have shown that tubular vesicles are favored when the growth of a compartment's membrane occurs more rapidly than its inner volume [112] (Fig. 4D). In this case, the surface area to volume ratio quickly increases leading to tubular vesicle morphologies. Upon mild mechanical agitation, the tubular vesicles divide into multiple daughter cells. This cycle containing growth and division can continue as long as membrane material, e.g. fatty acid micelles, is provided to the system. A similar system has been reported for bilayers adhering to elastic organosilicon substrates. If an initially stretched surface with an adhered lipid bilayer on it, is relaxed, lipid tubes spontaneously emerge from the membrane plane [132]. These examples depict that lipid membrane tubes can form conveniently, even in the absence of proteins and chemical energy. In this context, lipid nanotubes have recently become an integral part of a new hypothesis on protocell formation, growth, replication and division [133]. When lipid reservoirs were placed on mineral-like solid substrates, they autonomously underwent a series of topological transformations [113]. This process starts by spreading from the reservoir onto the substrate as a circular double bilayer membrane. The distal membrane (upper with respect to the surface) ruptures and spontaneously forms nanotubes. Some fractions of the nanotubes swell over time and transform into spherical ampiphilic compartments which can encapsulate compounds from the ambient solution and maintain them, a feature attributed to primitive protocells [113,134]. The resulting system is a population of surface-adhered protocells connected via a network of lipid nanotubes (Fig. 4E). The protocells can, in principle, transport water-soluble constituents, for example nucleotides or RNA fragments, which has, however, not yet been explicitly demonstrated in the described system. However, given the known transport properties of nanotube networks, genetic content can likely be transferred from one protocell to another, establishing a possible pathway to replication [133]. It was shown that upon application of a gentle hydrodynamic flow, the mature protocells can separate from the population and migrate to remote locations.

Understanding how eukaryotic cells evolved from prokaryotes gives a deeper understanding of current cell biology. The evolutionary aspects can also directly be important to medicine; a concrete and well known example is the antibiotics which target enzymes in bacteria also affecting the mitochondria, since they contain similar enzymes [135]. In the same way, understanding how bacteria form and employ nanotubes to communicate and transport matter can impact the understanding of the functionality of nanotubes in contemporary cells. The lipid nanotube as a ubiquitous biological structure is the example showing how simple mechanical membrane instabilities can be used by all forms of life and drive evolution. Proteins shape the membranes of current cells, in evolution it might have been more of a two way interaction, since nanotubes are spontaneously forming in any fluid membrane system, these nanotubular structures could have served as templates for evolving tubularprotein structures. Studies focusing on the model protocell formation and dynamics at the mesoscale show how a range of relatively simple mechanical and physicochemical processes can create nanotubular structures without the need for regulation. This suggests that the nanotubes could have existed even at the origin of life.

Open access funding provided by NTNU Norwegian University of Science and Technology (incl St. Olavs Hospital - Trondheim University Hospital). I.G. gratefully acknowledges the Research Council of Norway (Forskningsrådet), Project Grant 274433, UiO: Life Sciences

Convergence Environment, the Swedish Research Council (Vetenskapsrådet), Project Grant 2015-04561, as well as the startup funding provided by the Centre for Molecular Medicine Norway (RCN 187615), and the Faculty of Mathematics and Natural Sciences at the University of Oslo. P. D. gratefully acknowledges the Department of Physics, NTNU for support. We thank Dr. Aldo Jesorka from Chalmers University of Technology for comments and discussions.

**Open Access** This is an open access article distributed under the terms of the Creative Commons Attribution License (https://creativecommons.org/licenses/by/4.0/), which permits unrestricted use, distribution, and reproduction in any medium, provided the original work is properly cited.

**Publisher's Note** The EPJ Publishers remain neutral with regard to jurisdictional claims in published maps and institutional affiliations.
